# Comparison of Bioelectrical Impedance Parameters in Patients With Knee Osteoarthritis: A Case-Control Study

**DOI:** 10.7759/cureus.96766

**Published:** 2025-11-13

**Authors:** Udayan Das, Abhay Tyagi, Akshay Maharia, Barsha B Parida, Ganesh Rajesh Pande, Vedaant Parekh, Debashish Mishra, Ipsita Debata

**Affiliations:** 1 Department of Orthopaedics, Kalinga Institute of Medical Sciences, Bhubaneswar, IND; 2 Department of Physiology, Kalinga Institute of Medical Sciences, Bhubaneswar, IND; 3 Department of Community Medicine, Kalinga Institute of Medical Sciences, Bhubaneswar, IND

**Keywords:** bia, bioelectrical impedance analysis, impedance ratio, knee osteoarthritis, phase angle

## Abstract

Introduction: Osteoarthritis is the most prevalent type of arthritis, often significantly affecting the knee. Timely identification of knee joint osteoarthritis may facilitate the early implementation of preventive strategies and therapeutic measures, perhaps mitigating the course and the extent of the condition. Established methodologies, such as X-ray, computed tomography scan, magnetic resonance imaging, and arthroscopy, possess limitations and typically do not identify early-onset knee joint osteoarthritis. This study aimed to compare specific bioelectrical impedance parameters, including phase angle, impedance ratio, reactance, and resistance, between patients with knee osteoarthritis and age- and sex-matched controls and to evaluate their diagnostic performance in identifying osteoarthritic changes.

Methodology:The study was an age- and sex-matched case-control study. The study participants were patients between the ages of 40 and 80 years diagnosed with knee osteoarthritis. The controls were patients without having osteoarthritis. Patients with inflammatory or rheumatic disease, any infection, endocrine-related arthropathy, secondary post-traumatic osteoarthritis, and hip osteoarthritis are excluded. A multifrequency bioelectrical impedance analysis (BIA) analyzer (version 4/20; QuadScan 4000, Bodystat Ltd., Douglas, Isle of Man, British Isles) was used to perform bioelectric impedance assessment.

Results: A total of 116 participants were enrolled in the study, with 58 patients with knee osteoarthritis as cases and 58 healthy controls. The mean reactance in the case group was 79.96 ± 27.89 ohms compared to only 54.68 ± 23.42 in the control group. This difference was statistically significant (p < 0.001). Although resistance was observed to be higher in the control group, it was not statistically significant (p = 0.134). The impedance ratio and phase angle were significantly higher in patients with knee osteoarthritis compared to healthy controls (p < 0.001). Predictive accuracy was highest for the phase angle, with an area under the curve (AUC) of 0.793, with sensitivity and specificity of 81% each.

Conclusion: The study findings demonstrate that the BIA parameters, including phase angle, were significantly different between the osteoarthritic knee and the normal knee.

## Introduction

Osteoarthritis is the most prevalent type of arthritis, often significantly affecting the knee. It is predominant in adults, increasing sharply after 40 years, and causes chronic pain and immobility. The prevalence of osteoarthritis in people more than 30 years of age has risen to about 14.8% in the last few decades [1]. The number increases with age, making osteoarthritis a significant contributor to global disability burden, with the knee being the most commonly affected joint [2].

In India, osteoarthritis is seen to have increased by almost 40 million cases (from 23.46 million to 62.35 million individuals suffering from osteoarthritis within a span of 29 years) [3]. Obesity is a modifiable risk factor for knee osteoarthritis. Excess weight causes increased joint stress and accelerates cartilage breakdown. Globally, obesity rates have doubled since 1980, with projections indicating rising prevalence [4]. Apart from obesity, stress, genetic predispositions, race, advanced age, female sex, prior trauma, and hormonal imbalances have been identified as risk factors for the development of knee osteoarthritis [5-7].

Timely identification of knee joint osteoarthritis may facilitate the early implementation of preventive strategies and therapeutic measures, perhaps mitigating the course and the extent of the condition. Diagnosis during the advanced stage renders interventions less effective, as there is a distinctive change in the structural characteristics of the synovial cavity and articular cartilages. Numerous diagnostic techniques have been established or are under development; nevertheless, they lack reliability, cost-effectiveness, and simplicity in diagnosing early osteoarthritis. Established methodologies, such as X-ray, computed tomography (CT) scan, magnetic resonance imaging (MRI), and arthroscopy, are employed for diagnosing knee joint osteoarthritis [8-11]. Although ultrasonography (USG) is comparatively safe, cost-effective, and efficient in terms of time, it is very reliant on the technician's skills and is limited in evaluating deeper articular tissues due to acoustic shadowing [11]. Nonetheless, these approaches have limitations and typically fail to either identify early-onset knee joint osteoarthritis or impose higher costs, limiting their use. A novel detection technology is required for the early diagnosis of osteoarthritis to facilitate timely interventions for patients at high risk of disease progression.

It was proposed that biological cells, comprising cell membranes and intracellular fluids (ICF), are suspended in fluid and exhibit frequency-dependent behavior in response to different electrical stimuli [12,13]. Biological cells and tissues generate a complicated bioelectrical impedance when subjected to electrical excitation [13]. Bioelectrical impedance analysis (BIA) of tissues is performed by applying an electric current of varying frequencies to different tissues, as tissues exhibit varying conductive and resistive properties. Phase angle (PhA) is an index derived from BIA. PhA assesses nutritional status by reflecting water distribution and body cell mass. It is determined by two parameters: resistance, related to body water, and reactance, which represents the cells' ability to store energy. PhA is considered a nutritional status marker in various clinical conditions, with higher values indicating healthier lean tissue [14]. However, limited studies have been performed to determine its utility. BIA is a comprehensive, non-invasive, safe, straightforward, portable, compact, and affordable diagnostic assessment technology that offers a valuable methodology, particularly suited for economically disadvantaged populations.

Therefore, the present study was designed to (1) compare bioelectrical impedance parameters such as PhA, impedance ratio, reactance, and resistance between patients with knee osteoarthritis and matched controls and (2) assess the diagnostic accuracy of these parameters in differentiating osteoarthritic knees from normal knees.

## Materials and methods

Study design and setting

An age- and sex-matched case-control study was conducted by the Department of Orthopedics in collaboration with the Department of Physiology at Kalinga Institute of Medical Sciences in Bhubaneswar, Odisha, Eastern India. The study was conducted between July 2025 and September 2025.

Sample size calculation

Based on a case-control study conducted by Ertürk et al. [15], where the mean PhA in cases and controls was 10 ± 4.8 and 7.6 ± 3.9, respectively, the minimum sample size was calculated to be 108 (54 in each osteoarthritis group and control group), with a power of 80%, an alpha value of 0.05, and a 95% confidence interval. Sample size was calculated using Stata version 19.5 (StataCorp, College Station, TX, USA).

Study participants

The case group consisted of patients between the ages of 40 and 80 years who were diagnosed with knee osteoarthritis and attended the outpatient department (OPD) of the Department of Orthopedics, as well as admitted patients in the Department of Orthopedics who were scheduled for total knee replacement.

The control group consisted of patients with arthroscopically confirmed normal cartilage and without osteoarthritis, matched with cases for age (±2 years) and sex.

Patients with inflammatory or rheumatic disease, any infection, endocrine-related arthropathy, secondary post-traumatic osteoarthritis, or hip osteoarthritis were excluded from the study. The Kellgren-Lawrence Classification System was used to classify a patient with or without knee osteoarthritis based on the X-ray findings [16].

**Figure 1:**
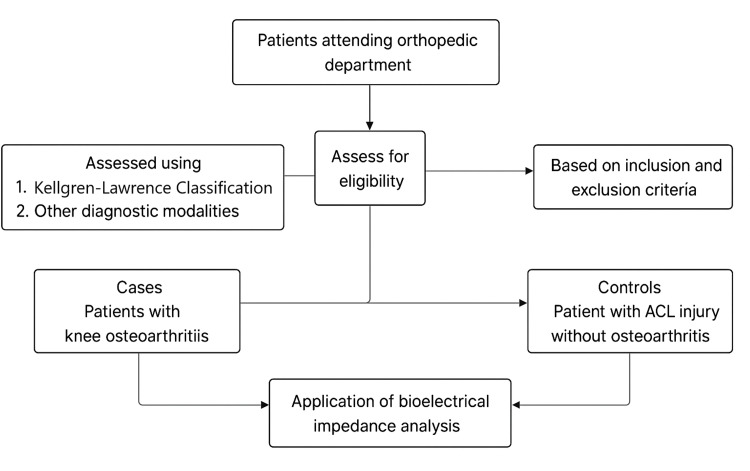
Flowchart showing the recruitment of the study participants ACL: anterior cruciate ligament

The flowchart depicting the recruitment of the study participants is shown in Figure 1.

Study procedure

All the study participants were informed in detail about the study and the procedures. Written informed consent was obtained from each subject before enrolment in the study. General demographic characteristics of the study participants will be collected, along with their anthropometric measurements and current physical activity status. The physical activity of the enrolled subjects was assessed using the self-reported International Physical Activity Questionnaire (IPAQ), a 12-item scale with a reliability of 80% [17]. The subjects were then referred to the Department of Physiology for bioelectric impedance assessment. A multifrequency BIA analyzer (version 4/20; QuadScan 4000, Bodystat Ltd., Douglas, Isle of Man, British Isles) was used to perform bioelectric impedance assessment. In the supine position, recordings were performed in a quiet environment with an ambient temperature of 22-24°C. The BIA analyzer was calibrated before recording to produce consistent results.

During the assessment, participants were asked to rest flat with arms and legs spread slightly and rest for 20 minutes. The skin of the dorsal side of the right hand and right foot was cleaned using 70% ethanol. Self-adhesive disposable electrodes (four in number) were attached to this area, and leads were connected to the electrodes and attached to the device. A low-intensity current will be passed through the body. Values of parameters such as resistance, reactance, and impedance ratios were subsequently obtained and analyzed. A derived indicator called PhA was also calculated.

Statistical analysis

Data was compiled and analyzed using IBM SPSS Statistics for Windows, V. 27.0 (IBM Corporation, Armonk, NY, USA). Categorical variables were expressed in frequency and percentage. Continuous variables were interpreted as mean and standard deviation. The distribution of continuous variables was assessed using the Shapiro-Wilk test. The comparison between the two groups was performed using the Mann-Whitney U test for continuous variables and the chi-squared test for categorical variables. The predictive accuracy of bioelectrical impedance parameters in distinguishing between knee osteoarthritis and other conditions was assessed using a receiver operating characteristic (ROC) curve, which determined the area under the curve (AUC). The cutoff value was calculated using Youden's index, and the sensitivity and specificity were also calculated at this cutoff. A p-value of <0.05 was taken as statistically significant.

Ethical consideration

The study was approved by the Institutional Ethics Committee of Kalinga Institute of Medical Sciences (approval number: KIIT/KIMS/IEC/2281/2025).

## Results

A total of 116 participants were enrolled in the study, with 58 patients with knee osteoarthritis as cases and 58 healthy controls. The mean age of cases was 48.98 ± 15.60 years, which was similar to that of the controls (48.02 ± 14.29 years). The male-to-female ratio in both groups was identical, indicating that the age and sex matching performed in the study was appropriate. The baseline characteristics of the cases and controls have been depicted in Table 1.

**Table 1:** Comparison of the baseline characteristics between cases and controls The chi-squared test (χ²) was used for categorical values; p-values of <0.05 were considered statistically significant SD: standard deviation; BMI: body mass index

Characteristic	Cases (n = 58)	Controls (n = 58)	P-value
Age (years) (mean ± SD)	48.98 ± 15.60	48.02 ± 14.29	0.949
Sex (n (%))			
Male	38 (65.5)	38 (65.5)	χ² = 0.000 (1.000)
Female	20 (34.5)	20 (34.5)	
Height (cm) (mean ± SD)	162.36 ± 9.65	158.21 ± 9.42	0.026
Weight (kg) (mean ± SD)	60.60 ± 9.56	62.17 ± 12.89	0.442
BMI (kg/m²) (mean ± SD)	23.07 ± 3.85	24.73 ± 4.11	0.029
Physical activity level (n (%))			
Medium/high	24 (41.4)	21 (36.2)	χ² = 0.327 (0.567)
Low/very low	34 (58.6)	37 (63.8)	

The bioelectrical impedance comparison between cases and controls is given in Table 2. The mean reactance in the case group was 79.96 ± 27.89 ohms compared to only 54.68 ± 23.42 in the control group. This difference was statistically significant (p < 0.001). Although resistance was observed to be higher in the control group, it was not statistically significant (p = 0.134). The impedance ratio and PhA were significantly higher in patients with knee osteoarthritis compared to healthy controls (p < 0.001).

**Table 2:** Comparison of the different bioelectrical impedance indicators between cases and controls The Mann-Whitney U test was used to compare cases and controls; p-values of <0.05 were considered statistically significant; and data are presented as mean ± SD SD: standard deviation

Indicators	Group		Total (mean ± SD)	U-value	P-value
	Case (mean ± SD)	Control (mean ± SD)			
Third space water (liters)	2.066 ± 1.67	1.74 ± 1.60	1.90 ± 1.64	1548.0	0.245
Resistance (ohms)	445.76 ± 150.43	487.46 ± 141.48	466.61 ± 146.89	1427.5	0.134
Reactance (ohms)	79.96 ± 27.89	54.68 ± 23.42	67.32 ± 28.61	894.0	<0.001
Impedance ratio	0.20 ± 0.10	0.13 ± 0.09	0.16 ± 0.10	812.0	<0.001
Phase angle in degrees	11.57 ± 4.74	7.45 ± 4.83	9.51 ± 5.20	785.0	<0.001

The third space water showed a significant negative correlation with age in both the case and control groups (p < 0.05). The bioelectrical impedance parameter, including PhA, did not show any significant correlation with age in either the case or control groups, except for resistance in the control group, which showed a significant correlation with age (p = 0.003) (Table 3).

**Table 3:** Correlation of age with bioelectrical impedance parameters between cases and controls r: Pearson correlation coefficient; p < 0.05 considered statistically significant

Indicators	Cases		Controls	
	Correlation coefficient (r)	P-value	Correlation coefficient (r)	P-value
Third space water in liters	-0.393	0.002	-0.267	0.041
Resistance in ohms	-0.044	0.740	0.378	0.003
Reactance in ohms	-0.062	0.644	-0.015	0.910
Impedance ratio	-0.165	0.217	-0.131	0.324
Phase angle in degrees	-0.244	0.065	-0.159	0.229

**Figure 2:**
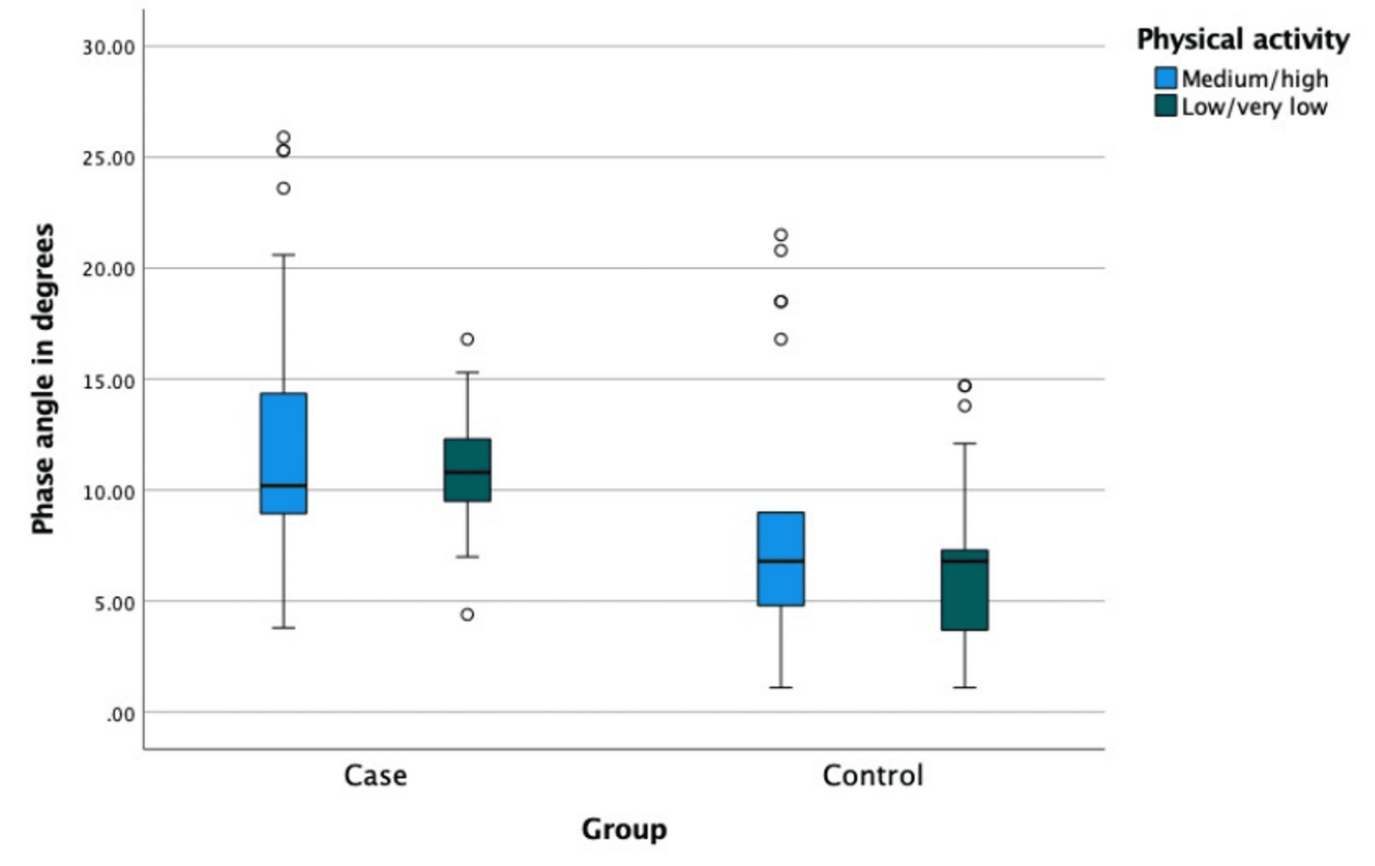
Phase angle with respect to physical activity between cases and controls

Regarding physical activity, there was no significant difference in PhA between medium to high activity and low to very low activity (p > 0.05) (Figure 2).

**Figure 3:**
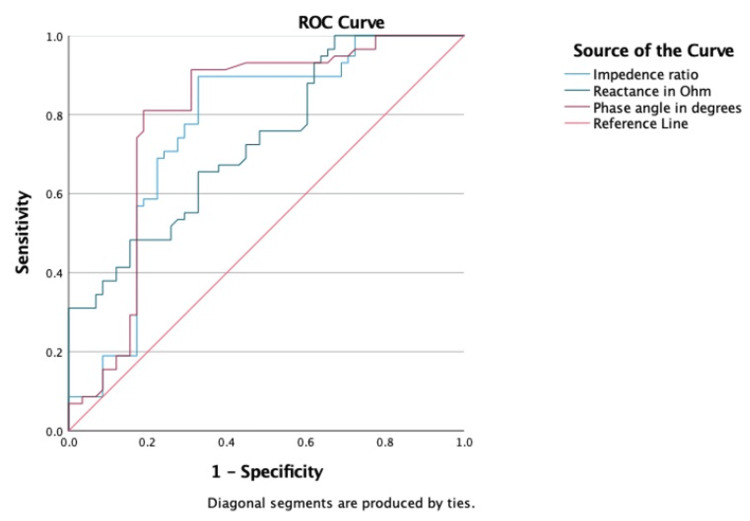
ROC curve of the bioelectrical impedance parameters for the diagnosis of knee osteoarthritis ROC: receiver operating characteristic

The diagnostic performance of bioelectrical impedance parameters was evaluated using ROC analysis (Figure 3). At a cut-off value of 8.75 (p < 0.001), PhA had the highest discriminating capacity of all the measures, with an AUC of 0.793 and 81% sensitivity and 81% specificity. The corresponding odds ratio showed a strong and statistically significant association with disease status, at 4.27 (95% CI: 2.47-7.38). The impedance ratio also demonstrated good diagnostic performance (OR = 3.24 (2.16-4.84); AUC = 0.762; sensitivity = 89.7%; specificity = 67.2%; p < 0.001). The diagnostic accuracy of reactance was found to be fair (AUC = 0.731; sensitivity = 65.5%; specificity = 67.2%; p < 0.001; OR = 1.98 (1.31-2.98)). Conversely, resistance demonstrated a low capacity for discrimination (AUC = 0.419; p = 0.134; OR = 1.30 (0.85-2.00)). Out of all the factors that were investigated, PhA and impedance ratio were identified as the most reliable predictors (Table 4).

**Table 4:** Diagnostic performance of bioelectrical impedance parameters ROC curve analysis of bioelectrical impedance parameters for differentiating cases and controls. The cut-off values were determined using Youden's index ROC: receiver operating characteristic; AUC: area under the ROC curve; CI: confidence interval; OR: odds ratio

Parameters	Cut-off	AUC	Sensitivity	Specificity	P-value	Odds ratio (95% CI)
Resistance	279	0.419	89.7%	17.2%	0.134	1.30 (0.85-2.00)
Reactance	65.35	0.731	65.5%	67.2%	<0.001	1.98 (1.31-2.98)
Impedance ratio	0.127	0.762	89.7%	67.2%	<0.001	3.24 (2.16-4.84)
Phase angle	8.75	0.793	81%	81%	<0.001	4.27 (2.47-7.38)

## Discussion

The articular cartilage of the knee joint is an avascular, aneural, and active tissue consisting of a matrix composed of collagen and proteoglycans with unevenly scattered cells. In healthy cartilage, tissue fluid constitutes up to 80% of the overall weight. Inorganic ions, including calcium, magnesium, sodium, chloride, and potassium, are present in the fluid phase and possess a constant charge density. Motion at the knee joint produces a stress-relaxation reaction as pressure exerted during motion displaces the ionic synovial fluid laterally, resulting in alterations in electrical conductivity. Osteoarthritis of the knee joint due to various etiologies promotes wear and tear, forming pits, crevices, and microclefts. Along with it, inflammatory mechanisms exacerbate the breakdown of the cartilage matrix and the severity of osteoarthritis. All these changes can be detected through changes in electrical activity, resistance, and reactance [18-21]. 

Our study observed a significant difference in BIA between healthy controls and patients with knee osteoarthritis, including PhA. The predictive ability of the PhA was best among all the bioelectrical parameters in distinguishing knee osteoarthritis from a knee without osteoarthritis. Distefano et al. investigated the impact of mechanical tourniquets on limb circulation during ischemia and edema using electrical impedance, concluding that impedance analysis is an efficient, simple, and straightforward technique for monitoring tourniquet application durations and assessing both preventive strategies and therapeutic interventions [22]. Alvarenga and Souza evaluated human osteoarthritis with healthy knee participants, revealing a substantial difference (p < 0.01), and proposed that bioelectrical impedance may be utilized to identify and assess inflammatory pathological states of the knee joint [23]. Subsequently, Neves et al. investigated osteoarthritis and demonstrated that bioimpedance is a sensitive method for assessing the physiological alterations linked to osteoarthritis, proposing that the bioimpedance methodology can facilitate the diagnosis of knee osteoarthritis [24].

Bioelectrical impedance operates on the principle that distinct tissues exhibit varying conductivity and resistance characteristics at various intensities of electric current. Few publications have asserted that BIA can conduct swift electrical measurements and assess body weight [25,26]. Resistance correlates with body water, as increased water is retained in lean body mass. A greater value signifies healthier lean tissue. Reactance refers to the capacity of cells to store energy, which is associated with bodily capacitance. Moreover, bioelectrical impedance can be employed to detect changes in bodily fluid volume, as bodily fluids serve as conductors [27,28]. The number of bodily fluids is crucial in the elderly, as these fluids facilitate the transport of energy and oxygen to skeletal muscles and essential organs, including the brain, heart, and lungs. Consequently, to reduce the expenses associated with medical examinations and promote a healthier lifestyle, the implementation of BIA for regular screening would be advantageous in caring for the senior population. Identifying osteoarthritis in its pre-clinical phase might give physicians a window of opportunity to take preventive intervention.

The study's strength lay in its case-control design, which effectively addressed the most significant confounding factors, such as age and sex, in relation to knee osteoarthritis. We observed a significant difference in body mass index between the groups, which may affect the outcome. However, it was noted that the control group had a higher body mass index and this difference is primarily due to height differences. Therefore, it can be assumed that the difference in BMI may not significantly affect the outcome.

Limitation

Since it is a case-control study, it cannot prove a causal or temporal link between the development of osteoarthritis and bioelectrical impedance parameters. Impedance measurements may be impacted by potential confounders that were not controlled for, such as dietary intake, circadian fluctuations, comorbid conditions (such as diabetes or hypertension), and differences in physical fitness or hydration status. It is advised that these findings be confirmed and expanded upon in future multicentric longitudinal studies involving larger and more varied populations. 

## Conclusions

This study found that patients with knee osteoarthritis differed significantly from age- and sex-matched controls in bioelectrical impedance parameters, especially PhA and impedance ratio. These results suggest that BIA could be a straightforward, non-invasive supplement to musculoskeletal evaluation and may represent physiological alterations associated with osteoarthritic disease.

The persistent group-level differences demonstrate the potential clinical relevance of BIA as a supportive screening tool, even though the current case-control design only permits associative interpretation. To confirm these results and determine the prognostic and diagnostic efficacy of BIA in knee osteoarthritis, more multicentric and longitudinal investigations using standardized methods are necessary.
